# Clinical Quantitative Analysis of Proteids in Stomach Contents1Read at the thirty-third annual meeting of the Medical Association of Central New York, held at Rochester, N.Y., October 16, 1900.

**Published:** 1901-04

**Authors:** A. L. Benedict

**Affiliations:** Buffalo, N.Y.


### CLINICAL QUANTITATIVE ANALYSIS OF PROTEIDS IN STOMACH CONTENTS.[1]

By A. L. BENEDICT, Buffalo, N. Y.

IN THE examination[2] of stomach contents, thus far we have gone all around the real function of the stomach, without testing as to how well or how poorly that function was performed. We have learned how much hydrochloric acid remained in excess of that taken up by food, whether a similar excess of ferments was present, how much the stomach had interfered with starch digestion, when the stomach passed its contents on to the small intestine, but we have ignored the direct issue of the amount of albumin transformed into albumoses and true peptones.

The method demonstrated to you today consists in the successive precipitation of the proteids in solution in the stomach contents and their approximate measurement, by centrifugalising the three precipitates, acid albumin, albumoses and peptones. At first thought, this would seem to be a very simple matter, but I can assure you that it required a large amount of research, including interviews and correspondence with chemists, and of laboratory experiment, to place it on a practical,clinical basis. Strangely enough, no analytic chemist seems to have undertaken the problem before. Any physiologic chemistry contains directions regarding the reactions of the various forms of nitrogenous matter, but, in practically every case, it was assumed that an unlimited supply, usually prepared artificially, was available. In all instances, the tests were given with the implicit understanding that the investigator would perform them as a matter of scientific curiosity and not with the practical, analytic object of separating and quantitating the ingredients of a mixed mass of proteids. For several years, it has been my custom to take up some special problem, either in physical diagnosis, applied chemistry of digestion, microscopic technic or some other theoretic topic that seemed likely to yield practical results if properly applied, and to make a winter's study of it. But the problem of proteid digestion in the stomach has occupied two winters, simply because my ignorance of certain scientific details of chemistry compelled me to grope in the dark while, on the other

1. Read at the thirty-third annual meeting of the Medical Association of Central New York, held at Rochester, N.Y., October 16, 1900.

2. Dr. Benedict gave a demonstration of his original centrifugal method of quantitating proteid digestion in stomach contents, for which he was awarded the medal of the American Medical Association at its meeting in 1900. Dr. Brown, of Rochester, assisted in the tests.

hand, lack of familiarity with the condition of medical practice, prevented chemists from giving me exactly the information which would have been of the greatest use. I mention this point only to urge a more general coöperation between the medical scientist and the medical practitioner, in attacking the many problems that lie before us and whose solution will make medicine more and more an applied science, as well as an art.

One of the difficulties in the way of careful analysis of chyme is the small amount obtainable—not usually over eighty and often less than thirty cubic centimeters, after the ordinary test meals. Free HCL and total acidity can be estimated at one titration, if we are careful and meet with no mishap. Combined acidity requires another titration, proteids another and at least a small quantity must be reserved for various qualitative tests. While the tests for acidity are best applied to unfiltered chyme, proetolysis requires a clear filtrate and a considerable loss occurs on account of the mass left on the filter. As a matter of practical experience, I have found that the tests for proteids must be made with ten or sometimes only five cubic centimeters. Filtration, especially if much mucus is present in the stomach contents, is a tedious process. I have tried all sorts of expedients, such as the use of absorbent cotton, separation by a colander, and the like, but have not succeeded as yet in obtaining rapid filtration. By centrifugalising the stomach contents, they can be readily separated into three layers, the lower one consisting of undigested food, the upper one of butter and mucus, the middle one of comparatively clear liquid. By removing the upper layer with a swab, the middle one can be decanted and filtered in the usual way, without the hours' delay required if the stomach contents are simply poured into the filter.

To save your time, I have brought with me ten cubic centimeters of filtered stomach contents. Acid albumin is thrown down by boiling and, after cooling, is centrifugalised. You can see the firm white sediment of albumin reaching up to the second mark on the centrifuge tube. This represents 2 per cent. of firmly packed albumin by volume, equivalent, as I have found by actual drying and weighing, to about a quarter or fifth as much of dry albumin. This is the normal maximum.

There is no natural separation of the various steps of the peptonising process but, by common consent, chemists consider that everything proteid not precipitated by ammonium sulphate in saturated solution is a peptone, and that everything between albumin and peptone may be called albumose. Of course, the process of peptonisation could be further subdivided by using different reagents. To precipitate albumose—or rather albumoses—I add one gram of ammonium sulphate to the ten cubic centimeters of decantate, dissolve the salt by heat and cool. As the mixture cools, you will notice that a turbidity forms, due to albumose. This is very light and is precipitated only with the greatest difficulty; in fact, I do not usually try to clear it absolutely by the centrifuge, but simply estimate what is thrown down by 10,000 revolutions. Here we have a mere trace which is normal, although 1 per cent. may be taken as the normal maximum.

To precipitate peptones, I employ phosphomolybdic acid, which makes a very bulky precipitate. I should prefer tannic acid, the precipitate from which is only about a sixth as bulky and tannic acid, is the reagent usually recommended by chemists, even in experimenting with stomach contents, but they forget that tannic acid also precipitates starch, which is almost invariably present in chyme. This little oversight alone cost me several months' time, as it necessitated throwing out quite a series of observations. You note that the precipitate with phosphomolybdic acid amounts to two cubic centimeters in ten. Normally, this precipitate is from 10 to nearly 30 per cent. of the filtered chyme. This relatively enormous bulk suggests that something else than peptones is precipitated, and it was only after careful search of chemic literature and consultation with chemists that I became convinced that we could rely on this reagent. Phosphomolybdic acid precipitates alkaloids and certain biliary constituents, but it is impossible that there should be anything of a nonproteid nature in the filtrated chyme, in sufficient quantities to interfere with the result desired. For instance, to show that ordinary saline matters and waste that might be present in the stomach could not cause a precipitate, we need only add phosphomolybdic acid to urine, when we find a reassuring absence of any precipitation.

You will ask what practical result we can derive from such an examination. The method is comparatively simple and by it we can tell exactly how much digestive work the stomach is accomplishing. In general, we shall find an excess of lower forms of proteids in cases of subacidity and deficient formation of ferments. For instance, in cancer, we should expect at least 2 per cent. of acid albumin, probably as much albumose and only 5 or 10 per cent. of peptone, by bulk. We must also bear in mind that an excess of an end-product may mean either usually good digestion or poor absorption.

In the present instance, the total acidity was 96°, and the free hydrochloric acidity 34°, which are within normal limits. The case is one of mild gastric dilatation, but at present, perhaps due to stimulation of function by strychnine and reinforcement by the administration of hydrochloric acid, digestion is progressing normally.

